# Elucidating nanostructural organization and photonic properties of butterfly wing scales using hyperspectral microscopy

**DOI:** 10.1098/rsif.2024.0185

**Published:** 2024-09-11

**Authors:** Anna-Lee Jessop, Primož Pirih, Limin Wang, Nipam H. Patel, Peta L. Clode, Gerd E. Schröder-Turk, Bodo D. Wilts

**Affiliations:** ^1^ School of Mathematics, Statistics, Chemistry and Physics, Murdoch University, Perth, Western Australia 6150, Australia; ^2^ Department of Chemistry and Physics of Materials, University of Salzburg, Salzburg 5020, Austria; ^3^ Marine Biological Laboratory, University of Chicago, Woods Hole, MA 02543, USA; ^4^ Centre for Microscopy, Characterisation and Analysis, University of Western Australia, Perth, Western Australia 6009, Australia; ^5^ School of Biological Sciences, University of Western Australia, Perth, Western Australia 6009, Australia; ^6^ Research School of Physics, The Australian National University, Canberra, Australian Capital Territory 2601, Australia

**Keywords:** *Erora opisena*, structural colour, gyroid, chitin, nanostructure growth, index matching

## Abstract

Biophotonic nanostructures in butterfly wing scales remain fascinating examples of biological functional materials, with intriguing open questions with regard to formation and evolutionary function. One particularly interesting butterfly species, *Erora opisena* (Lycaenidae: Theclinae), develops wing scales that contain three-dimensional photonic crystals that closely resemble a single gyroid geometry. Unlike most other gyroid-forming butterflies, *E. opisena* develops discrete gyroid crystallites with a pronounced size gradient hinting at a developmental sequence frozen in time. Here, we present a novel application of a hyperspectral (wavelength-resolved) microscopy technique to investigate the ultrastructural organization of these gyroid crystallites in dry, adult wing scales. We show that reflectance corresponds to crystallite size, where larger crystallites reflect green wavelengths more intensely; this relationship could be used to infer size from the optical signal. We further successfully resolve the red-shifted reflectance signal from wing scales immersed in refractive index liquids with varying refractive index, including values similar to water or cytosol. Such photonic crystals with lower refractive index contrast may be similar to the hypothesized nanostructural forms in the developing butterfly scales. The ability to resolve these fainter signals hints at the potential of this facile light microscopy method for *in vivo* analysis of nanostructure formation in developing butterflies.

## 1. Introduction

**Figure 1.**
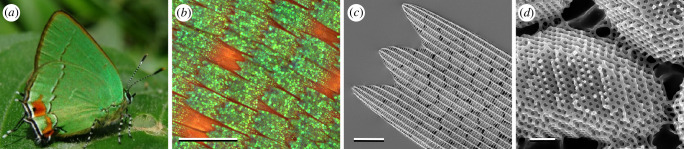
The green wings of *Erora opisena* originate from wing scales containing photonic gyroid nanostructures. (*a*) Photograph of *E. opisena*, reproduced with permission from P. Brodkin. (*b*) Light microscopy image of the wings of *E. opisena*. (*c*) Scanning electron microscopy (SEM) image of a single green scale showing the ridges, cross-ribs and discrete gyroid crystallites within the scale lumen beneath. (*d*) Gyroid crystallites imaged from the underside of the scale with the lower lamina removed. Scale bars: (*b*) 100 μm, (*c*) 10 μm and (*d*) 1 μm.

Some of the most visually stunning colour patterns in nature are created by structural colours. Structural colour arises as a result of the interference of light with periodic nanostructures and is known to occur across almost all kingdoms of life including animals [1–4], plants [5–7], bacteria [8] and protists [9,10] (for reviews, refer to [11–14]). Among those, butterfly wings are arguably the most well-recognized example of structural colour in nature. Butterflies employ a diverse set of nanostructural morphologies within their wing scales that behave as photonic structures [4,13]. The single gyroid crystals found in numerous green butterflies [15–20] are one of the more intriguing nanostructural morphologies, comprising a highly ordered, chiral and sponge-like network with cubic crystal symmetry [21] (figure 1). In butterflies, these gyroid photonic crystals reflect green light that probably functions in camouflage, as an aposematic signal, and in mate choice. Biophotonic single gyroid structures have also been reported in weevils [22] and birds [23] with slightly shorter and longer reflectance wavelengths compared with butterflies, respectively.

The development of butterfly nanostructures occurs during pupation. Each wing scale develops from a single epithelial cell that secretes a chitinous cuticle that follows the template of the cell’s plasma membrane [24,25]. Based on Ghiradella’s seminal ultramicroscopic studies [26–28], the prevailing hypothesis of gyroid formation in butterflies is that the wing scale cell’s smooth endoplasmic reticulum adopts a gyroid morphology and acts as a template to the nascent chitin polymer [16,18,27,29,30], while the whole scale cell is still living and containing cytosol. In part due to the inability to observe nanostructure growth in butterflies *in vivo*, several aspects of this formation or growth process remain open or speculative.

The optical properties of single gyroid photonic crystals are well understood, through band structure analyses [31–34] and numerical reflectance calculations using finite-difference time-domain (FDTD) methods [31,34,35], plane-wave expansions [16,32,36] or scattering matrix methods [37]. Single gyroid optical properties have also been analysed through experiments on custom fabricated three-dimensional gyroid geometries on the centimetre scale [38,39] or nano/micrometre scale [40–42], refer to [43] for a review. The optical observations in gyroid biophotonic crystals in butterflies [16,19,20,34,44] or replicas thereof [45] are largely explained as photonic crystal effects, with or without pigmentary absorption. It is well known that the immersion of dielectric photonic crystals in media with refractive indices different from that of air (*n* = 1) changes the colour of their reflectance and can be used in sensor applications [46–48]; the change in coloration of butterfly wings immersed in alcohol or other liquids is a popular demonstration experiment [49]. If a chitin gyroid structure were present in the pupae during development, it would be immersed within the cytosol ( 1.36<n<1.39 , [50]) leading to a change in reflected colour compared with the dry state. Indeed, the transition of some green butterflies from a bronze colour state just after emerging to a bright green coloration is likely to be due to this effect [51].

The focus of this study is on the green wing scales of the butterfly, *Erora opisena* (formerly *Thecla opisena*; figure 1). *Erora opisena* is a small Lycaenid butterfly with an overall size of approximately 2  cm, native to the northern Neotropics [18]. The ventral sides of both wings of the butterfly are vivid green, with the hindwings containing small red and white patches (figure 1*a*). The green colour arises from wing scales that contain single gyroid photonic nanostructures within the scale lumen (refer to [18, fig. 1] and figure 1*b–d*). Remarkably, these single gyroid structures form disjoint crystallites with a pronounced size gradient along the scale [18] (a similar crystallite structure has also been observed in *Mitoura grynea* [27]). Gyroid crystallites towards the tip of the scale are larger and gradually decrease in size towards the base of the scale. Wilts *et al*. [18] hypothesized that this size gradient may be a developmental sequence frozen in time and that the formation of the crystallites could be through a growth or extrusion process. If this were the case, one would expect the gyroid photonic crystals to begin developing initially as small crystallites towards the tip of the wing scale, growing larger over time. In this sense, the optical properties of the smaller gyroid crystallites found towards the base of the adult wing scales may be analogous to those of crystallites at an early stage of development.

Here, we present a novel application of a hyperspectral microscopy (HSM) method, and use auxiliary scanning electron microscopy (SEM), X-ray tomography and optical modelling, to explore the relationship between gyroid crystallite size and reflectance and the effect of immersing wing scales in refractive index liquids with refractive indices similar to cytosol. HSM is a microscopy technique that provides spectral data for each image pixel [52]. This differs from conventional light microscopy that only records in three colour channels (red, blue and green; RGB), and from multi-spectral imaging that records in 3–15 colour channels. HSM, on the other hand, can record in many hundreds of colour channels, depending on the HSM set-up (here, we record from 30 channels). HSM also differs from the more conventional microspectrophotometry (MSP) that also records a full spectrum but can only record from a single location at a time, whereas HSM allows spectra to be recorded from many locations simultaneously. This advantage enables efficient reflectance measurements to be collected across a large area, such as an entire wing scale. As such, HSM is a method that combines the spatial resolution of light microscopy with good spectral resolution, as an alternative to MSP.

Hyperspectral microscopy, and hyperspectral imaging more broadly, has been applied across many different fields (refer to the reviews in [53,54]) providing a method for measuring spectra across small spatial scales. To date, HSM has already been used to elucidate colour generation in adult butterfly wings [55], for species discrimination in *Drosophila* [56], coating quality control [57], *in vivo* measurements of human irises [58], for angular-dependent reflectance of iridescent butterflies [59], for determination of screening pigments in the eye [60,61] and to measure defects in self-assembled cellulose nanocrystals [62].

While HSM, like all conventional light microscopy methods, cannot resolve the biophotonic nanostructures, HSM can resolve the optical reflections the structure creates. By combining this method with imaging techniques that can resolve nanostructural features (SEM and X-ray tomography) and optical modelling, optical reflections measured using HSM can be linked to specific nanostructures and their structural properties. This article demonstrates that the HSM method is sufficiently sensitive and accurate to resolve finer ultrastructural details of the photonic signal of butterfly wing scales. This includes resolving signals in situations (namely in immersion experiments) that mimic the situation one may find in a developing wing scale, hence pointing at the potential of HSM for future applications in imaging biophotonic nanostructural development *in vivo*.

## 2. Results

**Figure 2.**
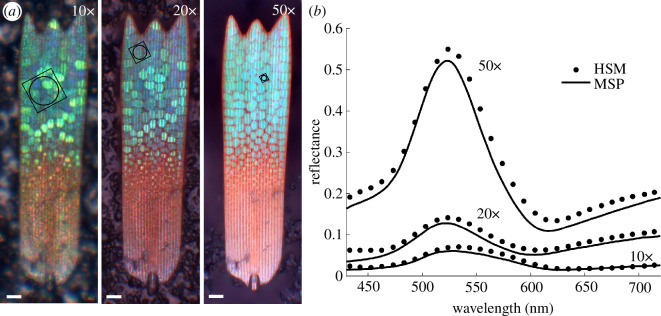
Reflectance spectra from small patches of a single green wing scale from *E. opisena* measured using HSM and MSP. (*a*) Light microscopy images of a single wing scale taken with 10× (numerical aperture (NA) = 0.30), 20× (0.60) and 50× (0.95) objectives, showing locations of measurement areas producing the results shown in (*b*). The colour of crystallites changes with the NA of objectives. (*b*) Reflectance spectra from each location in (*a*) measured using HSM (dots) and MSP (lines). The scale bars in (*a*) are 10 μm, the circle sizes represent the measurement area for MSP, the square sizes represent the measurement area for HSM (10 000 camera pixels).

Single green wing scales from *E. opisena* show a diminishing gradient of green coloration from the scale tip to the middle, whereas the lower half is red-coloured and no photonic nanostructure is present (figure 2*a*). When attached to the wing, the wing scales partially overlap and the reddish parts are mostly hidden (figure 1*b*).

Observing the scale through microscope objectives with increasing numerical apertures (NAs) (10×, NA = 0.30; 20×, NA = 0.60; 50×, NA = 0.95), the green colour of crystallites changes in hue, desaturates, and increases in intensity and uniformity (figure 2*b*). A similar NA-dependent effect, which is due to illuminating the sample and collecting the light from ever larger spatial angles, has been previously observed in the gyroid structures of the butterfly, *Callophrys rubi* [63]. Using HSM and MSP, we measure reflectance (spectra) as a measure of the relative reflectance to a mirror standard, refer to §4.

We used the green, red and white scales of *E. opisena* to compare the spectra obtained with HSM and MSP methods (refer to figure 2*b* and electronic supplementary material, figure S1). HSM was performed between 420 and 720 nm with a wavelength resolution of 10 nm (refer to §4), while MSP was performed between 380 and 760 nm. To directly compare the two methods, the same locations and approximately equal measurement areas were used (refer to figure 2*a*). The MSP measurement areas were approximately 320, 78, 14 µm^2^ for the 10×, 20×, 50× objectives, respectively. The corresponding HSM square measurement area (100 × 100 camera pixels) could in principle be further reduced to 10 × 10 or less pixels, achieving a diffraction-limited measurement area less than 1 µm with a 50× objective.

The reflectance curves obtained with HSM (dots) and MSP (lines) were closely matching for green, red and white *E. opisena* wing scales (figure 2*b*; electronic supplementary material, figure S1). The peak wavelength for green scales measured by HSM with 10×, 20× and 50× objectives occurred at 533, 523 and 523 nm, respectively, differing by less than 1% from MSP measurements (528, 521 and 523 nm, respectively). Reflectance amplitudes also agreed well, differing on average over all wavelengths by 16%, 20% and 23%, respectively. For the red and white scales, the MSP and HSM reflectance measurements differed by 4% and 1%, respectively (electronic supplementary material, figure S1).

As further validation, we reproduce the previous finding that the green wing scales of *E. opisena* contain a blue-absorbing pigment [18], through the HSM analysis of absorbance, rather than reflectance, in scales immersed in refractive index liquids matched to chitin, as described in electronic supplementary material, figure S6.

### 2.1. Crystallite size determines reflectance amplitude

**Figure 3.**
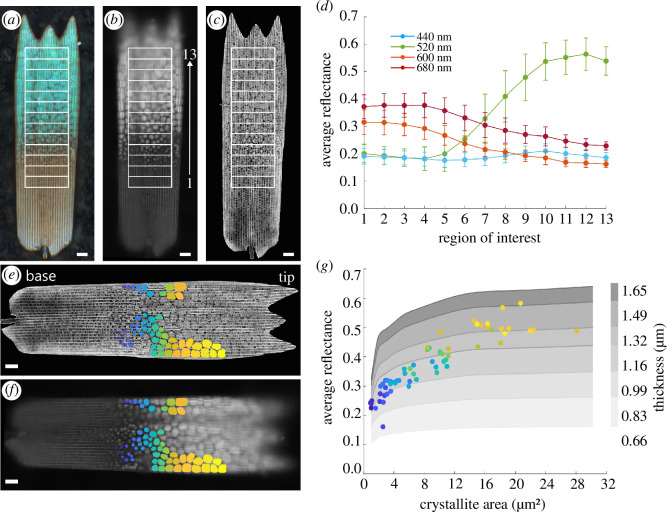
Reflectance increases with crystallite size across a single-wing scale. (*a–c*) Microscopy images of a single green *E. opisena* wing scale showing the locations of 13 regions of interest that were used to measure relative reflectance from the base to the tip of the scale. (*a*) Colour image taken under white light illumination. (*b*) Monochromatic image at 520 nm. (*c*) SEM on-view image of the same scale. The crystallites are visible below the array of ridges and cross-ribs. (*d*) Average reflectance (±s.d.) across 13 regions of interest measured using HSM. Coloured points show the average reflectance under 440 nm (blue), 520 nm (green), 600 nm (orange) and 680 nm (red) light. (*e,f*) The locations of segmented crystallites from a single scale superposed on the SEM image (*e*) and the monochromatic light microscopy image (under 520 nm illumination). (*g*) The average reflectance of 520 nm light across each segmented crystallite versus the area of each crystallite. The colours of each point correspond to the colours of each segmented crystallite in panels (*e*) and (*f*). Grey shading shows the theoretical reflectance obtained from FDTD simulations of single gyroid geometries with a refractive index equal to 1.56, a lattice parameter of 330 nm, a solid volume fraction of 0.3, areas between 1 μm^2^ and 30.25 μm^2^ and thicknesses between 0.66 and 1.65 μm.

The lumen of *E. opisena* green scales contains discrete gyroid crystallites that exhibit a pronounced size gradient with smaller crystallites towards the middle and larger crystallites at the tip [18] (figure 3*a–f*). HSM enabled us to efficiently investigate whether there is a relationship between crystallite size and reflectance. For this, we measured the average reflectance across 13 regions of interest (200 × 800 pixels) beginning from the base of the scale (where no photonic nanostructure is present) and ending at the tip of the scale (where the largest crystallites are located) (figure 3*a–c*). The reflectance at 520 nm increased from the base (approx. 0.20) to the tip (approx. 0.55) of the scale. The opposite trend was observed at 600 and 680 nm, where the reflectance was higher towards the base of the scale. Reflectance at 440 nm remained approximately constant across the scale length.

Segmentation of individual crystallites was based on the SEM image (figure 3*e*). This allowed the identification of the exact locations of the structures without the uncertainty caused by the optical effects of the structures. We further analysed only the crystallites that appeared sharp in the HSM image stack, which also happened to be smaller crystallites that were smaller in area. In total, 62 crystallites were segmented, ranging in size from 0.9 to 28 µm^2^, with smaller crystallites displayed in blue and larger crystallites displayed in yellow (figure 3*e,f*). The mean reflectance at 520 nm was calculated for each crystallite (dots in figure 3*g*, the colours consistent with figure 3*e,f*). The mean reflectance of crystallites increased with their size and ranged from approximately 0.16 for the smaller crystallites to approximately 0.58 for the larger crystallites (figure 3*g*). A statistical Pearson’s correlation analysis yields a moderate, positive correlation between crystallite area and reflectance, with statistical significance (*r*(60) = 0.55, *p* < 0.05).

The measured trend is supported by optical modelling of idealized chitin gyroid structures. We modelled the reflectance of idealized chitin gyroid structures with thicknesses varying between 0.66 and 1.65 µm, and areas varying between 1 and 30.25 µm^2^. The optical modelling showed that reflectance increased with crystallite area (grey shading in figure 3*g*); however, increasing only the area does not fully explain the experimental results. The optical modelling shows that both crystallite area and thickness determine the absolute reflectance of the crystallites (grey shading in figure 3*g*). This hints that in the green scales of *E. opisena*, the larger crystallites at the tip of the scale could be approximately 50% thicker than the smallest crystallites towards the scale base, based on the readings 1.3−1.5 and 0.8−1.1 µm for large and small crystallites, respectively (grey shading in figure 3*g*).

To further investigate this, we measured the thickness and area of gyroid crystallites from an X-ray tomogram of a different *E. opisena* wing scale (electronic supplementary material, figure S3). Crystallites that covered a larger area were indeed also thicker, with the largest measured crystallites (area approx. 40 µm^2^) having a thickness of approximately 2.5 µm and the smallest measured crystallites (area approx. 3 µm^2^) having a thickness of approximately 0.5 µm (electronic supplementary material, figure S3). These data are consistent with thickness estimates that can be extracted from the experimentally measured reflectance and optical modelling data presented in figure 3*g*.

Finally, absorbance measurements, carried out on scales immersed in index-matched oil, are consistent with the statistical observation that crystallites that are larger in area are also thicker (electronic supplementary material, figure S6). We show that absorbance (at 450 nm wavelength) increases with crystallite area with a remnant statistical variation in absorbance for crystallites of the same size; both are consistent with the reflectance data in figure 3*g*. These data appear consistent with an even spatial distribution of the pigment within the chitin body of the gyroidal nanostructure. A correlative study comparing spatially registered HSM absorbance, HSM reflectance and SEM images of the same scale could further support the above observation on a crystallite-by-crystallite basis.

### 2.2. Reduced refractive index contrast causes dimmed, red-shifted reflectance

Photonic crystals in mature insect scales are usually composed of a network of dry, chitinous cuticle and a network of air [1,15,16,18], resulting in a refractive index contrast between the two networks of approximately 1.55 [64]. The situation is quite different in the growing scales, where the refractive index contrast is much lower, since one network is cytosol with a refractive index 1.36<n<1.39 [50] and the other is probably hydrated chitin, n<1.55 . To mimic these conditions, we can replace the air in the network with a fluid of known refractive index. We therefore used HSM to measure the reflectance of isolated wing scales immersed in different refractive index liquids.

**Figure 4.**
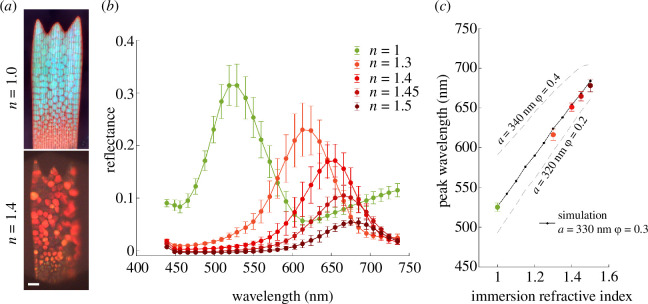
Change in reflectance spectra of *E. opisena* wing scales upon immersion in refractive index matching oils. (*a*) Light microscopy images of a green wing scale in air (*n* = 1.00, upper panel) and immersed in oil with refractive index *n* = 1.40 (lower panel). (*b*) Reflectance curves of 10 crystallites (mean ± s.d.) from a single green wing scale in air (green) and from scales immersed in oils with refractive indices of 1.30 (orange), 1.40 (light red), 1.45 (medium red) and 1.50 (dark red). Measurements for each crystallite were averaged from areas containing 1600 camera pixels. (*c*) The peak reflectance wavelength (mean ± s.d.) for each immersion experiment (coloured points, mean ± s.d.) along with the simulated peak reflectance wavelengths determined for theoretical immersion refractive indices of 1 to 1.50 (black and grey lines). FDTD simulations were conducted on a single gyroid geometry within a range of values for the lattice parameter a and volume fraction ϕ . The combination of a=330nm and ϕ=0.3 , which provides a close agreement with the measured data, fits with estimates from electron microscopy (black points and line). The combinations a=340nm,ϕ=0.4 and a=320nm,ϕ=0.2 are provided for reference (grey dashed lines). The refractive index used for the chitin phase is 1.56.

When immersed in a medium with a refractive index greater than 1.30, single green scales appear predominantly red (figure 4*a,b*). HSM measurements across 10 crystallites from each scale show a red shift of approximately 90 nm in the mean peak reflectance wavelength between scales measured in air (mean peak wavelength = 526 nm) and scales measured in an immersion oil (refractive index liquid) of refractive index 1.30 (mean peak wavelength = 616 nm; figure 4*b,c*). Increasing the refractive index further red-shifted the peak to mean peak wavelengths of 651, 664 and 678 nm for oil with refractive indices of 1.40, 1.45 and 1.50, respectively (figure 4*b,c*). Using FDTD simulations, we modelled the reflectance of three different idealized gyroid crystals of chitin in different refractive index environments (grey dashes and black lines and points in figure 4*c*). The three models were gyroid crystals with lattice parameter equal to 320, 330 and 340 nm, with solid volume fractions of 0.2, 0.3 and 0.4, respectively. The model comprising the gyroid crystal with lattice parameter 330 nm and chitin volume fraction φ=0.3 produced reflectance values that most closely matched our experimentally measured mean reflectance and were within 1 s.d. of these values.

## 3. Discussion

### 3.1. Gaining structural insight from hyperspectral microscopy and other optical measurements

The beautiful and unique arrangement of gyroid crystallites in *E. opisena* provides a useful example for the relationship between structural features and the optical signal they cause. It, therefore, enables a discussion of the advantages and limitations of using optical measurements to infer structural features or parameters.

Specifically for *E. opisena*, the discrete and variably sized gyroid crystallites offer an opportunity to gain insights into the formation of these remarkable structures [18]. In line with the findings from Wilts *et al.* [18], we have here shown that the crystallite areas are largest towards the tip (approx. 40 µm^2^) of the scale and smallest towards the base (approx. 3 µm^2^), using both SEM (figure 3) and X-ray tomography (electronic supplementary material, figure S3). Using HSM, we have also shown that a positive correlation exists between the crystallite area and the intensity of reflected green wavelengths (figure 3*g*). Full-wave optical modelling further supported these observations but also hinted that, in addition to the crystallite area, the thickness of the crystallites plays a role (figure 3*g*).

An analysis of X-ray tomography data showed that larger crystallites (in terms of area) were also thicker (electronic supplementary material, figure S3) and both the area and thickness values obtained from the tomography data corresponded to the optical model parameters used to generate reflectance data that corresponded to our experimentally measured values (figure 3*g*). By comparing our tomography measurements with the optical modelling results (figure 3*g*), we show that it is possible to infer the thickness of each crystallite from their reflectance, and suggest that the larger crystallites at the scale tip are also thicker. Further investigations, such as a correlative tomography and spectroscopy study, would help to clarify this observation and enable more accurate predictions of crystallite sizes based on reflectance.

More broadly, it is interesting to ponder what knowledge about a nanostructure can be obtained from the optical signal it generates. It is clear that the optical signal does not provide an unambiguous signature of the structural form. A striking example of that is provided by the green wing scales of *Parides sesostris* and *P. aeneas bolivar* that use a gyroid nanostructure and a multi-layer structure, respectively, to generate green colouration. In combination with pigmentation, these two different structural forms generate indistinguishable reflectance spectra [65]. An additional example with relevance to our study species comes from the Lycaenid *Chrysozephyrus aurorinus* that uses a perforated multi-layer to generate a similar green coloration to that of *E. opisena* [66]. Furthermore, in a recent review of structural colour in butterflies, Thayer & Patel highlight several different structural forms that can be tuned to generate the same peak wavelengths [4].

While optical methods, including the HSM method applied here, cannot provide an unambiguous identification of structure, they can be very useful for the analysis of changes to structural parameters such as volume fraction, lattice parameters, dielectric contrasts, sample thicknesses, crystallite sizes, orientations, etc. This is illustrated in electronic supplementary material, figure S5, which shows, through optical modelling, that changes to these structural parameters result in differences in reflectance that depend on the parameter that is varied. The idea to extract structural parameters from optical measurements is not new and, for example, is realized when ellipsometry is used to analyse film thicknesses or refractive indices of thin films [67]. It has also been realized in the context of biological nanostructures, for example, in the development of structural colour in leaf beetles [68] and in the mechanisms of reversible colour change in beetles (reviewed in [69]).

By studying the optical response, and similar to our approach taken in figures 3 and 4, it should be possible to gain information about changes of structural or material parameters. Such changes could be evolution with time, or analysis of spatial variations across a sample, or indeed could be experimental parameters such as light direction as in iridescence studies.

### 3.2. Elucidating biophotonic nanostructure development using immersion experiments

Our current understanding of biophotonic nanostructural development in butterflies is mostly based on studies using either fixed pupal wing tissue from discrete time points or from adult wing scales [26,27]. Electron microscopy studies of fixed pupal wing tissues have offered insights into some of the more complex nanostructural formations such as the formation of lamella ridges and the growth of single gyroid photonic crystals [16,26,27]. More recently, studies using confocal microscopy have uncovered details of the cellular dynamics of scale formation including the role of F-actin in the formation of scale fingers and ridges [70,71], in the spacing of ridges [72], and in the formation of the honeycomb lattice found in the upper lamina of some papilionid butterfly species [73].

Many details of the developmental processes underlying nanostructure formation in butterflies remain elusive and can only be uncovered through continuous, spatio-temporally resolved *in vivo* investigations of living scale cells. However, imaging nanostructural features *in vivo* remains an unsolved challenge. Constrained by Abbe’s diffraction limit, the spatial resolution of light microscopy is limited to hundreds of nanometres, a resolution too low to directly observe the development of photonic nanostructural features. In the last two decades, several super-resolution microscopy techniques utilizing fluorescent probes have emerged, enabling *in vivo* imaging with resolutions as low as 50  nm (for a review, refer to [74]). While these techniques are promising, non-toxic fluorescent probes are scarce and expensive, and the strong illumination required by these techniques can easily disturb delicate subcellular processes, particularly when imaging must be performed over a span of several hours or days. Recently, a label-free imaging technique using optical speckle-correlation reflection phase microscopy achieved a resolution high enough to resolve some of the larger nanostructural changes, showing ridge and lamellae formation *in vivo* [75]; however, the technique is not able to achieve the resolution needed to resolve the smaller gyroid nanostructures.

An alternative approach, suggested by this paper, is to take advantage of the fact that these nanostructures behave as photonic structures. As this article has shown, it is possible to infer information about crystal size (figure 3), and refractive index contrast (figure 4) from the optical signals that are created by photonic nanostructures. Importantly, our results suggest that HSM may be a convenient method for studying the small optical signals one would expect to occur during the formation of biological photonic crystals *in vivo*. While MSP can provide similar spectral information, the HSM technique that we have applied in this study allows accurate measurements of spectra at the spatial resolution limit of optical microscopy while also efficiently providing spatial information (figure 2). For *in vivo* imaging, this spatial information will be a necessity as keeping measurement consistency may be difficult during the inevitable movements caused by larval growth and HSM enables spatial registration of an image series.

Specifically, by immersing adult wing scales in refractive index liquids with similar refractive index to cytosol ( 1.36<n<1.39 [50]) (figure 4), we attempted to mimic the refractive index conditions that are likely to exist in a developing gyroid nanostructure. A reduction in the refractive index contrast between the chitin nanostructure and its surrounding medium causes a red shift and a reduction of the reflectance (figure 4*b,c*). We additionally simulated this scenario using optical modelling on three different idealized gyroid nanostructures with varying lattice parameters and solid volume fractions (figure 4*c*). Our simulations of a gyroid nanostructure with lattice parameter equal to 330 nm and a solid volume fraction equal to 0.3, resulted in a good match to our experimentally measured mean reflectance and was within 1 s.d. of the mean (figure 4*c*). Lattice parameter and solid volume fraction are not easily obtained with high accuracy from the SEM images, but the best fitting values from our optical models correspond to those values reported previously from a related species [17,18].

Whether the red shift also occurs in the developing butterfly remains to be seen, but these experimental simulations demonstrate the capacity of HSM to locally and accurately measure small reflectance signals. These results on mature wing scale nanostructures highlight that HSM has the appropriate spatial, temporal and wavelength resolution to study the optical signals expected in developing butterfly scales.

### 3.3. Hyperspectral microscopy as a tool to obtain spatially resolved reflectance

Hyperspectral microscopy is an established tool for obtaining spatially resolved spectral information. HSM creates a ‘data cube’ of two spatial dimensions and one spectral dimension; it does so by compiling a stack of microscopy images, each measured with monochromatic light of increasing frequency. This article has demonstrated the feasibility and usefulness of HSM for the analysis of mature insect nanostructures and its potential for future *in vivo* application to developing nanostructures.

**Figure 5.**
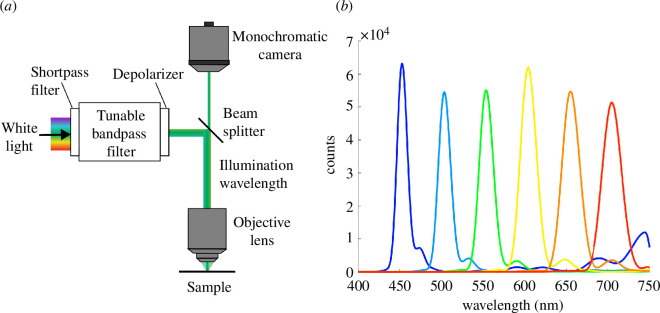
Hyperspectral microscopy set up and spectral output. (*a*) Schematic of the HSM consisting of a light source, a liquid crystal tunable bandpass filter, a monochromatic camera and an optical microscope (refer to §4 for details). (*b*) Intensity of incident broadband light after filtering with the liquid crystal filter for centre wavelengths of 450–700 nm in 50 nm steps measured using a spectrometer.

Our implementation of the HSM technique uses a monochromatic camera and a liquid crystal tunable filter. These two devices can be easily added to most standard optical microscopes (figure 5). Tunable filters have been used previously in a variety of contexts, for example, to rapidly resolve variations in electroluminescence of light-emitting diodes (LEDs) [76], to obtain simultaneous low-resolution spectrophotometry of multiple stars [77], for fine arts diagnostics [78], in fluorescence microscopy [79], and to analyse reflectance of butterfly wings [59]. Their ease of use and relatively low cost ($8000 USD) make them an attractive option for spectrally resolved microscopy, including for an *in vivo* experimental set-up.

Comparing HSM with MSP techniques, several advantages and limitations become apparent. HSM offers enhanced spatial resolution allowing access to smaller measurement spots (figure 3). HSM is inherently a spatially resolved method, with the spatial resolution given by the spatial resolution of the optical microscope. In contrast, spatial resolution in MSP can only be obtained by scanning the sample point by point, with the minimal measurement area (typically around 2 µm^2^) providing a limit on the resolution.

The superior spatial resolution of HSM comes with a trade-off of limited spectral range and reduced spectral resolution. The spectral resolution is generally not a problem, as reflectance spectra of biophotonic structures are usually quite smooth (refer to reflectance spectra in e.g. [4,11,12,16]). Particularly for ultraviolet–visible–near infrared (UV-VIS-NIR) spectral analysis and rapid data acquisition, a wavelength range extension, an increase in response speed and a reduction of the sidebands (figure 5*b*) would be welcome. The wavelength range of HSM can be matched to the transmission limit of microscope optics by using a more traditional combination of a broadband (XBO) source, and a monochromator or a gradient bandpass filter, but this also brings about the necessity of focus control due to chromatic aberrations [60,61]. Off-the-shelf multi-channel LED sources based on dichroic mirrors, or multi-emitter LEDs with adequate spatial homogenization are usable if the wavelength resolution is not of prime importance. Narrower spectra can be achieved by combining the LEDs through a diffraction grating [80]. Compared with these solutions, the liquid crystal filter technology is attractive because it is lightweight, transportable and can be easily installed both in the illumination and the observation path.

The recording time for the spatial-spectral data cube in HSM is given by the time it takes to step through the frequency bands and take one grey-scale microscopy image per step (the switching time of the liquid crystal filter of approximately 50 ms is negligible). Even for the slowest measurements at the lowest aperture, a full data cube took less than 30 s to record. While this is slower than the time for an MSP measurement in a single spot, it is faster than the time required to create a full spatial scan of MSP measurements.

The nature of the HSM data cube enables a convenient and flexible workflow. Having the spectral data associated with a microscopy image means that analyses such as region-of-interest analysis, slicing of data, registration to and correlation with other microscopy or image data, and statistical analyses can be carried out after the experiment. (Note that it is possible to reconstruct an RGB image from the HSM data cube and to calculate properties such as hue, desaturation, intensity and uniformity of coloration.)

Our implementation of the HSM technique and application to mature *E. opisena* wing scales support its potential for the future application to study the nanostructure development process in butterfly pupae. In terms of spatial and spectral resolution and in terms of its ability to measure these properties simultaneously and with sufficient sensitivity, the method is probably suitable for such studies. With a measurement time of less than 30 s, the temporal resolution should be sufficient to resolve many (biological) growth processes, albeit not sufficient for fast processes such as phase transitions; for temporal development studies, the ability to register (and hence) spatially correlate measurements taken from a typically slightly dynamic sample will be beneficial.

## 4. Methods

### 4.1. Samples

*Erora opisena* ([81]; Lepidoptera: Lycaenidae: Theclinae: Eumaeini) butterflies were caught in Chiapas, Mexico, and purchased from The Bugmaniac (https://www.thebugmaniac.com/). Individual wing scales were gently lifted off the wings using a cotton swab and placed onto a glass microscope slide or an aluminium stub covered with black aluminium foil (BKF12, T205, Thorlabs) or copper tape. Whole wings displayed in figure 1 were imaged using a Leica M205 C digital microscope. SEM images shown in figure 1 were imaged using a FEI Verios XHR scanning electron microscope after wing scales were sputter coated with platinum. The electron beam acceleration voltage was set to 5 kV and an in-lens secondary electron detector (Everhardt Thornley Detector) was used to image the scale at a working distance of approximately 5  mm.

### 4.2. Microspectrophotometry and hyperspectral microscopy

A modified Zeiss Axioscope 5 optical microscope was used to perform both MSP and HSM measurements. Microscope objectives included Zeiss EC Epiplan-Apochromat objectives with a magnification of 50× (NA = 0.95), 20× (NA = 0.60) and 10× (NA = 0.30) for the measurements of figures 2 and 3 and a multi-immersion objective Zeiss 25× (NA = 0.80) LD LCI Plan-Apochromat for the measurements in oils presented in figure 4.

For MSP, illumination was provided by a halogen light source (OSL2, Thorlabs Inc.) passed through a collimating lens (COP4-A, Thorlabs). Relative reflectance spectra were collected through a microscope sideport below the tube lens, with the light path consisting of a mirror, a focusing quartz lens and a 200 µm quartz fibre (FC-UVIR200, Avantes) attached to a spectrometer (AvaSpec-ULS2048XL-EVO, Avantes). The measurement spot diameter of 4, 10 or 20 µm using the 50×, 20× and 10× objectives, respectively, was set to the centre of the image using a mirror in the object plane. An aluminium mirror (PF10−03−F01, Thorlabs) served as the reference.

For HSM, epi-illumination for reflectance measurements was provided by a Zeiss Axioscope 5 microscope collimated white LED source or a halogen light source (OSL2, Thorlabs) that was passed through a collimating lens (OSL2COL, Thorlabs) and a shortpass filter (FESH0750, Thorlabs). The collimated light was passed through a tunable liquid crystal bandpass filter (Kurios, Thorlabs), and a quartz depolarizer to counteract any polarization effect of the liquid crystal filter (DPU−25, Thorlabs; a schematic diagram is shown in figure 5*a*). Transillumination for the absorbance measurements was provided by a Zeiss collimated white LED source. Absorbance A(λ) was derived from the measured transmittance T(λ) through $A(\lambda )=-{log}_{10}[T(\lambda )]$ of a wing scale immersed in a fluid of refractive index 1.56.

Transmitted and reflected light were measured with a 20 MP monochromatic camera with pixel size 2.4 µm (BFS-U3−200S6M-C, Teledyne FLIR) mounted to the camera port of the microscope. For transmittance measurements, the transmitted light was first passed through the liquid crystal filter. The tunable bandpass filter was controlled over the serial interface by custom software written in MATLAB 2023a (The MathWorks Inc., USA). The bandwidth was set to deliver a restricted spectrum of light with a full width at half maximum of approximately 18  nm between 430 and 720 nm in 10 nm steps (figure 5*b*). For each wavelength, an image was captured with the monochromatic camera. Because the illumination intensity (figure 5*b*) and the camera sensitivity were not equal across all wavelengths, a calibration run was initially conducted on a reference aluminium mirror (PF10-03-F01, Thorlabs) for reflectance measurements and through the microscope glass slide for transmittance measurements. The exposure times and gain values of the camera were adjusted such that the average pixel intensity of each image was approximately equal. This ensured that pixels were neither over- nor under-exposed for any one wavelength. Subsequently, the sample was measured using the calibrated exposure and gain values and the reflectance or absorbance at each wavelength were calculated. Reflectance was calculated by averaging the pixels in the area of interest, subtracting the dark background from the sample data, and then dividing the sample data by the reference mirror to obtain relative reflectance.

RGB images were taken on the same microscope using a 20 MP colour camera (The Imaging Source, DFK 38UX304) from different focal planes. Focus-stacked images were then created using Helicon Focus Pro (Helicon Soft Ltd, Ukraine). All data were further analysed using custom scripts written in MATLAB 2023a (The MathWorks Inc.).

### 4.3. Correlation between reflectance and gyroid crystallite size

To investigate the effect of crystallite size on reflectance we measured the average reflectance of regions of interest from scale base to tip and the reflectance of individual gyroid crystallites of differing sizes. A single scale was placed onto black foil attached to an aluminium stub and HSM was performed using the 50× objective. Subsequently, the same scale was sputter coated with a thin layer (approx. 5−8 nm thick) of gold using a Cressington Sputter Coater 108 auto (120 s, 40 mA, background pressure 0.08 mbar) and imaged with a Zeiss Ultra Plus 55 SEM. The electron beam acceleration voltage was set to 5 kV and an in-lens secondary electron detector was used to image the scale at a working distance of approximately 5.5  mm. SEM images were acquired at high magnifications across different sections of the scale and subsequently stitched together using Adobe Photoshop 2023. HSM and SEM data were then manually superposed. The regions of interest displayed in figure 3*a–c* each contain 160 000 camera pixels that were averaged to obtain reflectance spectra for each region. The regions of interest shown in figure 3*e,f* were defined from the SEM image using the Image Segmenter App in MATLAB 2023a.

To gain an understanding of how crystallite area (as viewed in the SEM image) relates to the thickness of the crystallite, we measured the areas and thicknesses of 25 crystallites from two X-ray tomograms of a green *E. opisena* wing scale. Details of the X-ray tomography data collection can be found by Wilts *et al.* [18], and the tomography datasets are available from the Dryad Digital Repository [82]. Briefly, an isolated wing scale was imaged using a Zeiss Xradia 810 Ultra X-ray microscope producing two tomography datasets with voxel sizes of 64 nm, one dataset of the base of the wing scale and a second of the tip of the wing scale. The datasets were visualized and stitched together in Dragonfly (v. 2022.1.0.1249; Object Research Systems, Montreal, Canada) and subsequent analyses were conducted on the stitched data. Area was measured manually on slices of the dataset using the free-hand drawing tool in Dragonfly (electronic supplementary material, figure S3), thickness was measured on two perpendicular slices using the measurement tool and the average of the two values was used as the crystallite thickness.

### 4.4. Immersion oil experiments

Single green wing scales were immersed in refractive index liquids (Series A, Cargille Laboratories Inc.) with nominal refractive indices of 1.30, 1.40, 1.45 and 1.50 at 589.3  nm at 25°C and covered by coverslip. The mean reflectance of each scale immersed in the particular oil for at least 5 min was obtained by averaging HSM measurements from 10 randomly selected gyroid crystallites. Each measurement area was a square containing 100 camera pixels. The reflectance measurements in figure 4*b* labelled with a refractive index equal to 1, were wing scales measured in air beneath a coverslip. The multi-immersion objective (Zeiss 25× (NA = 0.80) LD LCI Plan-Apochromat) set to the image in immersion oil was used for all measurements and an immersion fluid (*n* = 1.51) was placed between the objective and the coverslip.

### 4.5. Finite-difference time-domain modelling

Light scattering by the gyroid nanostructures was simulated with the three-dimensional FDTD method, using Lumerical 8.29 (Lumerical Solutions, Vancouver, Canada). The gyroid nanostructures of *E. opisena* were approximated by simulating voxelized single gyroid geometries in Houdini FX 19.5 (Side Effects Software Inc, Toronto, Canada) or through an idealized single gyroid network approximated by triply periodic minimal surface model from its level-set equation [83].

Gyroid geometries were set up in a rectangular simulation box with the two lateral directions, corresponding to [110] and [100], having periodic boundary conditions and the third (vertical) direction corresponding to the incident light direction was also a [110] symmetry direction having a perfectly matched layer (PML) boundary condition. The gyroid geometry that most closely matched our experimental data consisted of repeating unit cells with lattice parameter 330 nm and a solid volume fraction 0.3, resulting in the peak reflectance wavelength of 525 nm, approximately equal to that of our experimental reflectance measurements. The gyroid consisted of cuticular chitin with a refractive index of 1.56 [64], and we varied the refractive index of the surrounding medium between 1.00 and 1.55. Simulations of area and thickness dependence used PML boundary conditions on all boundaries of the simulation box that was adjusted in size to fully fit the gyroid geometry. Light, with wavelengths of 400–800  nm, was incident in normal direction onto the structure along the [110] direction.

A reasonable estimate for the lattice parameter a=330nm and the chitin solid volume fraction φ=0.3 of the gyroid was determined by the following method: the range of previously observed lattice parameters for gyroid geometries in butterflies [17,20,22,29], was discretized in 5 nm intervals. For each value for a , FDTD simulations for an air-chitin single-gyroid crystal were carried out by scanning the range of reasonable volume fractions to find the φ value for which the peak reflectance matched the peak reflectance observed in our experiments. For this set of pairs of (a,φ) , FDTD simulations were carried out where the n=1 hollow phase was replaced by n=1.5 . Out of these pairs, the pair (a,φ)=(330,0.3) was chosen as the one that gave the best match with the peak reflectance of the experiment with n=1.5 immersion oil. Further values of (a,φ)=(320,0.2),(340,0.4) were simulated to provide reference points.

## Data Availability

All data used for the analysis are available from the Dryad Digital Repository [82]. Supplementary material is available online [84].
